# Multivariable Regression Analysis in *Schistosoma mansoni*-Infected Individuals in the Sudan Reveals Unique Immunoepidemiological Profiles in Uninfected, egg+ and Non-egg+ Infected Individuals

**DOI:** 10.1371/journal.pntd.0004629

**Published:** 2016-05-06

**Authors:** Tayseer Elamin Mohamed Elfaki, Kathrin Arndts, Anna Wiszniewsky, Manuel Ritter, Ibtisam A. Goreish, Misk El Yemen A. Atti El Mekki, Sandra Arriens, Kenneth Pfarr, Rolf Fimmers, Mike Doenhoff, Achim Hoerauf, Laura E. Layland

**Affiliations:** 1 Institute of Medical Microbiology, Immunology and Parasitology (IMMIP), University Hospital of Bonn, Bonn, Germany; 2 Department of Parasitology and Immunology, College of Medical Laboratory Science, Sudan University of Science and Technology, Khartoum, Sudan; 3 Animal Resources Research Corporation, Ministry of Livestock, Fisheries and Rangelands, Khartoum, Sudan; 4 Institute of Medical Biometry, Informatics and Epidemiology (IMBIE), University Hospital of Bonn, Bonn, Germany; 5 School of Biology, University of Nottingham, Nottingham, United Kingdom; 6 German Centre for Infection Research (DZIF), partner site, Bonn-Cologne, Bonn, Germany; Leiden University Medical Center, NETHERLANDS

## Abstract

**Background:**

In the Sudan, *Schistosoma mansoni* infections are a major cause of morbidity in school-aged children and infection rates are associated with available clean water sources. During infection, immune responses pass through a Th1 followed by Th2 and Treg phases and patterns can relate to different stages of infection or immunity.

**Methodology:**

This retrospective study evaluated immunoepidemiological aspects in 234 individuals (range 4–85 years old) from Kassala and Khartoum states in 2011. Systemic immune profiles (cytokines and immunoglobulins) and epidemiological parameters were surveyed in n = 110 persons presenting patent *S*. *mansoni* infections (egg^+^), n = 63 individuals positive for *S*. *mansoni* via PCR in sera but egg negative (*Sm*PCR^+^) and n = 61 people who were infection-free (*Sm* uninf). Immunoepidemiological findings were further investigated using two binary multivariable regression analysis.

**Principal Findings:**

Nearly all egg^+^ individuals had no access to latrines and over 90% obtained water via the canal stemming from the Atbara River. With regards to age, infection and an egg^+^ status was linked to young and adolescent groups. In terms of immunology, *S*. *mansoni* infection *per se* was strongly associated with increased SEA-specific IgG4 but not IgE levels. IL-6, IL-13 and IL-10 were significantly elevated in patently-infected individuals and positively correlated with egg load. In contrast, IL-2 and IL-1β were significantly lower in *Sm*PCR^+^ individuals when compared to *Sm* uninf and egg^+^ groups which was further confirmed during multivariate regression analysis.

**Conclusions/Significance:**

Schistosomiasis remains an important public health problem in the Sudan with a high number of patent individuals. In addition, *Sm*PCR diagnostics revealed another cohort of infected individuals with a unique immunological profile and provides an avenue for future studies on non-patent infection states. Future studies should investigate the downstream signalling pathways/mechanisms of IL-2 and IL-1β as potential diagnostic markers in order to distinguish patent from non-patent individuals.

## Author Summary

Schistosome infections are a major public health problem and currently 230 million people are infected with these blood-dwelling parasitic helminths. Schistosomiasis remains the most prevalent parasitic disease in the Sudan and control of the infection relies on large-scale administration of praziquantel. Although treatment is immediately effective, it is not a cure and therefore as soon as individuals re-enter freshwater sources with infected vectors, they are at risk of being re-infected. Therefore, increasing access to clean drinking water and adequate sanitation are important measures to reduce the risk of schistosome infection. During this study people from endemic areas in the Sudan were classified according to the presence of *S*. *mansoni* DNA in sera or eggs in stool samples and all analysed with regards to epidemiological and immunological parameters. In addition samples from *S*. *mansoni* infection-free individuals from the same endemic regions were used as controls. Our findings suggest that epidemiological factors and immune responses to schistosomes depend on the actual infection status (patent versus pre-patent/low egg producing). This improves our understanding of the biology of the disease which facilitates the development of techniques to identify early stages of pathology (fibrosis) which could help prevent further damage and morbidity.

## Introduction

Schistosomiasis is elicited by parasitic trematodes and can lead to a chronic disease state. It remains one of the most prevalent neglected tropical diseases with an estimated 800 million people at risk and currently more than 230 million infected individuals [1–3]. The disease is widespread in tropical and sub-tropical areas, especially in poor communities without access to clean drinking water and adequate sanitation. Epidemiological surveys show that at least 90% of people requiring treatment for schistosomiasis live in Africa [4]. Humans become infected with schistosomes through skin penetration by cercariae that are released into fresh water by snail intermediate hosts. After a period of weeks, they mature into adult worms and produce fertilised eggs that are either shed into the environment through faeces or urine, depending on the infective species, or are retained in host tissues [5]. In freshwater, miracidia hatch from the eggs and infect the appropriate snail host [3]. The highest prevalence and intensities of infection occur in young adolescents, but prevalence can persist during adulthood especially in individuals who have frequent contact with freshwater sources during their daily activities such as obtaining drinking water, laundry, bathing, and fishing [3].

The three major schistosome species that parasitize man are *Schistosoma haematobium* (which causes urinary schistosomiasis) and *S*. *mansoni* and *S*. *japonicum* which inhabit blood vessels of the liver and intestine causing intestinal schistosomiasis [6]. In the majority of cases chronic infections are clinically silent although severe pathology can develop in a few individuals ranging from mild cercarial dermatitis to severe tissue inflammation which can lead to life threatening urogenital pathology or hepatosplenomegaly [7–9]. Interestingly, morbidity as a result of schistosome infection is not caused by adult worms [3] but arises from a granulomatous tissue reaction mediated by CD4^+^ T cell responses to eggs that become trapped in the liver, intestinal or urogenital tissues [5, 8]. The host’s immune response, generated against schistosome-specific antigens, e.g. schistosoma egg antigens (SEA), plays a critical role in both dictating the severity of tissue inflammation and associated disease [8]. The exact immunological outcome during schistosomiasis is dependent on the balance of Th2, Th1 and regulatory cells, and the complex immunological interplay of their secreted cytokines [10, 11]. After an initial schistosome-induced production of the Th1 cytokine (IFN-γ), Th2 cytokines such as IL-4, IL-5 and IL-13 are generated in response to established infections [5, 8, 12, 13]. It has also been shown that within the inflamed tissues there are pro-inflammatory cytokines such as TNF-α and IL-6 [14, 15]. More recently, research has shown that Th17 cells mediate the development of immunopathology during certain chronic helminth infections in humans, including schistosomiasis [9, 16] and such cells were also increased in the granulomas of *S*. *mansoni*-infected mice [9].

Schistosomiasis is a major health concern in most parts of the Sudan and leads to severe morbidity [11]. In fact, it is the most prevalent parasitic disease in this country with 24 million people at risk, 5 million recorded cases of infection and a prevalence rate of 20% (data refer to the country before the separation of South Sudan in 2011) [17, 18]. The Sudan has wide river basin areas with large areas of irrigated agriculture sectors alongside the banks of the rivers. These freshwater sources are heavily populated with schistosome-infected snails and therefore have affected the Sudanese for many centuries, especially in regions with major irrigation systems [17]. This retrospective study aimed at identifying potential markers of schistosome infection. Individuals from schistosome endemic areas of Kassala and Khartoum states were grouped into i) schistosome-egg positive in stool samples, ii) schistosome-egg negative but *S*. *mansoni* PCR-positive in sera or iii) schistosome-egg negative and *S*. *mansoni* PCR-negative. A panel of immune parameters (cytokines and immunoglobulins) were then measured in each individual and assessed using binary multivariable regression models with epidemiological covariates: age, gender, exposure, education, latrines, co-infection. Egg^+^ individuals were highly associated with no latrine access and as well as young and adolescent groups. Immunologically, *S*. *mansoni* infection *per se* was strongly associated with increased SEA-specific IgG4 but not IgE levels. IL-6, IL-13 and IL-10 were significantly elevated in patently-infected individuals and positively correlated with egg load. In contrast, IL-2 and IL-1β were significantly lower in *Sm*PCR^+^ individuals when compared to *Sm* uninf and egg^+^ groups which was further confirmed during multivariate regression analysis. Thus, these findings indicate that non-patent or low egg intensity infections have a unique immune profile (elevated SEA-specific IgG4 with low IL-2 and IL-1β) and future studies could concentrate on investigating the downstream signalling pathways/mechanisms of IL-2 and IL-1β to reveal potential biomarkers of disease/pathology.

## Methods

### Ethics and study population

Ethical clearance, including oral consent, for this study was approved by the Ministry of Health-Kassala State Department of Preventive Medicine Office of the anti-bilharzia and intestinal worms New Halfa City. Oral informed consent was obtained from all individuals and children were recruited only if consent was provided by a parent or legal guardian. Written consent was not required since a representative of the ministry of health was present for patient sampling at all times. All individuals completed a questionnaire regarding their health status, knowledge and living conditions and oral consent was noted by the examiner on these forms. In the questionnaire it was asked whether they had a latrine available at home, their level of education and potential exposure to schistosome infection. With regards to education either the current school year of the individual was determined or at what stage they left school (primary, secondary, higher education). The parameter "exposure" was based on the daily water source of the individual, here they were asked whether they acquired water from the canal, a pipe supply or a donkey cart (delivered). Those individuals obtaining water from the canal were classified as "exposed".

**Fig 1:**
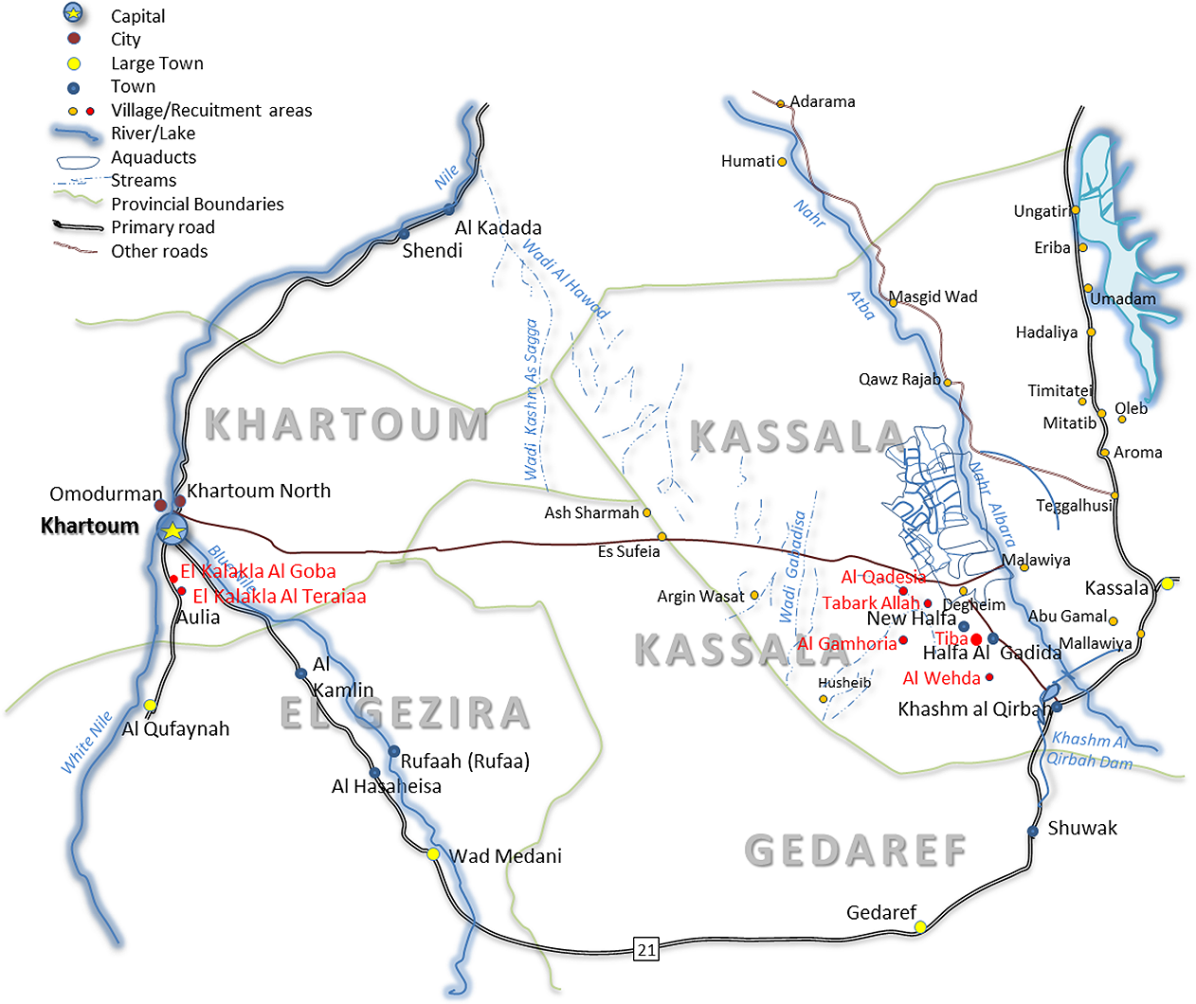
Map of study area. Schematic representation of the regions of Kassala and Khartoum states in the Sudan. Red circles denote villages in which study participants resided.

Participants were recruited from the area of New Halfa City in Kassala state, Sudan. This study area included the villages Al Gamhoria, Al Qadesia, Al Wehda, Tabark Allah and Tiba which are located approximately 1–2 km away from vector-residing water sources. Study participants were also recruited from the villages Al Kalakla Al Goba and Al Kalakla Al Teraiaa in Khartoum state located along the White Nile (Fig 1). Between March and October 2011, 770 individuals participated in this Ministry of Health survey and the distribution of this sampling is shown in S1 Fig. Of those 770 individuals 110 were *S*. *mansoni* egg positive in stool samples. In this retrospective study, we assessed a further 124 non-egg positive individuals from the same villages via *S*. *mansoni* PCR. Those individuals were then subdivided into *S*. *mansoni* uninfected (*Sm* uninf, n = 61) and *Sm*PCR^+^ egg-negative (n = 63). Table 1 shows details about the infection groups within the study cohort. The age of the study subjects ranged between 4–80 years (median 12 years). Overall, 55.98% of males and 42.02% of females participated in the study.

**Table 1:** Characteristics of study population.

		*Sm* uninf (n = 61)	*Sm*PCR^+^ (n = 63)	egg^+^ (n = 110)	p value
**Age (median + range)**		13 years [5–77]	12 years [6–70]	11 years [4–80]	P = 0.003
**Eggs/g (median + range)**		0	0	72 [24–960]	
**Gender**	male	38 (= 62.3%)	48 (= 76.2%)	45 (= 40.9%)	p<0.001
	female	23 (= 37.7%)	15 (= 23.8%)	65 (= 59.1%)	
**Co-infection**	yes	18 (= 29.5%)	16 (= 25.4%)	56 (= 50.9%)	p = 0.001
	no	43 (= 70.5%)	47 (= 74.6%)	54 (= 49.1%)	
**Access to latrine**	yes	25 (= 41.0%)	33 (= 52.3%)	16 (= 14.5%)	p<0.001
	no	36 (= 59.0%)	30 (= 47.6%)	94 (= 85.5%)	
**Water supply**	canal	41 (= 67.2%)	42 (= 66.7%)	100 (= 90.9%)	p<0.001
	water pipeline	3 (= 4.9%)	4 (= 6.3%)	10 (= 9.1%)	
	donkey carts	17 (= 27.9%)	17 (= 27.0%)	0 (= 0.0%)	
**Education**	no schooling	53 (= 86.9%)	52 (= 82.5%)	110 (= 100.0%)	p<0.001
	primary school	5 (= 8.2%)	9 (= 14.3%)	0 (= 0.0%)	
	high school	3 (= 4.9%)	2 (= 3.2%)	0 (= 0.0%)	

According to their diagnostic status, individuals were categorized as either *Sm* uninf (no eggs in stool and PCR^-^), *Sm*PCR^+^ (PCR^+^ but no eggs in stool), egg^+^ (Kato Katz positive). Table 1 shows the age, gender, egg load and co-infection status. Individuals were co-infected with one or more of the following: *Hymenolepis nana*, *Giardia lamblia* and *Entamoeba histolytica*. All individuals were negative for *S*. *haematobium*. See S1 Table for further details. In addition, access to latrines in house, the daily water source of the individual and schooling level are also shown. Absolute and percentage in brackets are shown for each category. p values denote statistical differences between the groups for that parameter tested by chi square test.

### Parasitological investigations

For the detection of *S*. *mansoni* eggs, stool samples from all individuals were analysed using the Kato-Katz technique [19, 20]. Three slides were prepared for each stool specimen and the infection/intensity was expressed as eggs/gram faeces. Participants were also screened for the intestinal parasites *Hymenolepis nana*, *Giardia lamblia* and *Entamoeba histolytica*. Participants were furthermore screened for *Taenia saginata* infections, but no tapeworm infections were observed. Individuals were also screened for the presence of *S*. *haematobium* eggs using urine samples and a sedimentation technique [6] (S1 Table). All samples were negative for *S*. *haematobium* eggs and for soil transmitted helminths such as *Ascaris lumbricoides*, *Trichuris trichiura* and hookworms.

### Preparation of serum samples

Approximately 5 ml of whole blood was drawn into Vacutainer tubes (BD Biosciences Heidelberg, Germany) containing no anticoagulant and then incubated for 5 hours in an upright position at room temperature to allow clotting. Serum samples were aspirated, aliquoted into cryotubes and stored at -80°C.

### DNA extraction and real-time PCR

Due to the small volume of available serum, DNA was extracted from 100 μl serum from all individuals using the QIAampDNA Blood MiniKit and the QIAcube fully automated sample preparation system (Qiagen, Hilden, Germany). The sample volume was adjusted to 200 μl using sterile Dulbecco’s PBS and the DNA extraction was then performed according to the manufacturer’s protocol for body fluids. To increase the total yield of DNA, samples were subsequently concentrated by ethanol precipitation and resuspended in a final volume of 8 μl. Each extraction included an extraction control containing 200 μl sterile Dulbecco’s PBS instead of serum. A sensitive genus-specific real-time PCR for *S*. *mansoni* species targeting a high-copy tandem repeat (GenBank: M61098.1) unit within the *Schistosoma* genome was performed. Amplification was performed on a RotorGene 6000 (Corbett Research, Sydney, Australia) using Qiagen’s HotStarTaq DNA Polymerase (Qiagen) for amplification. The following concentrations of primers and Taqman probe were used: 500 nM of each forward primer 5’-ATA TTA ACG CCC ACG CTC TC-3’ and reverse primer 5’-GAA TCC GAC CAA CCG TTC TA-3’, 25 nM Taqman probe 6-Fam-5’-TCC GTT CAG TGG TTT CGG AGA-3’-BHQ1. The mastermix included final concentrations of 4 mM MgCl_2_, 50 μM dNTPs (Peqlab, Erlangen, Germany) and 0.5 Units HotStarTaq and 2 μl DNA in a total reaction volume of 20 μl. The temperature profile was 15 min at 95°C followed by 45 cycles of 94°C for 10 sec and 58°C for 40 sec. Fluorescence data were acquired on the green channel at the end of the second step. The limit of detection was 1.3 x 10^−9^ grams with greater than 93% reaction efficient. Each PCR included a positive serum control, a negative serum control from European donors, an extraction control and a no template control. All samples were run in duplicate and a sample was defined as positive when both replicates of the tested sample were positive and all included controls except the positive controls were negative. To determine a cut-off C_t_ to distinguish infected from uninfected sera, Receiver Operating Characteristic (ROC) analysis was done using the egg positive samples as definitive positives (n = 97) and European controls and *S*. *haematobium* as negative samples (n = 8). From the ROC analysis, a C_t_ of >26 had 80% sensitivity and 75% specificity. We found no correlation between *Sm*PCR levels and egg counts in egg positive individuals.

### Cytokine detection

For cytokine detection in serum samples, a Th1/Th2/Th9/Th17/Th22 13plex Kit FlowCytomix (eBioscience, Frankfurt, Germany) was used. This bead-based analyte detection system measured levels of IFN-γ, IL-1β, IL-2, IL-4, IL-5, IL-6, IL-9, IL-10, IL-12 p70, IL-13, IL-17A, IL-22 and TNF-α. Samples were measured using the FACS Canto II (BD Biosciences). Data were analyzed with FlowCytomix Pro 3.0 software (eBioscience). In addition, IL-8 levels in serum samples were determined using the Human IL-8 ELISA Ready-SET-Go! (2nd Generation) Kit from eBioscience according to the manufacturer’s instructions.

### Analysis of immunoglobulins

Individual serum samples from the surveyed Sudanese population were analysed for levels of SEA-specific IgE and IgG4. In brief, 96-well polysorb plates (Nunc, Roskilde, Denmark) were coated overnight at 4°C with 50 μl/well of 5 μg/ml SEA (BioGlab Ltd, Nottingham, UK) diluted in PBS, pH 9.6. Plates were washed 3 times in washing buffer (PBS, 0.05% Tween 20, pH 7.2 [Sigma-Aldrich, Munich, Germany]) and once in 1x PBS. Plates were blocked with 200 μl/well blocking buffer (PBS/1%BSA) for one hour at room temperature (RT). Following an additional washing step, 50 μl/well of diluted serum (1:4,000 for IgG4 and 1:20 for IgE) was added and incubated overnight at 4°C. After a further washing step, 50 μl/well of biotinylated secondary antibodies (IgG4 1:15,000, Sigma-Aldrich, IgE 1:1,000, Southern Biotech, Birmingham, USA) were added and incubated for two hours at RT. The plates were washed with 1x PBS and incubated for 45 minutes at RT with 50 μl/well of streptavidin-horse radish peroxidase (Roche Diagnostics, Mannheim, Germany; 1:5,000). After the last wash, 50 μl/well substrate solution containing tetramethylbenzidine (Sigma-Aldrich) were added to the wells and incubated for 15 minutes, after which the reactions were stopped with 25 μl/well 2N H_2_SO_4_ (Merck KGAA, Darmstadt, Germany). Optical density was measured using a SpectraMAX ELISA reader (Molecular Devices, Sunnyvale, USA) with wavelength correction (450 nm and 570 nm). Data were analysed with SOFTmax Pro 3.0 software (Molecular Devices). In serum samples from healthy European donors SEA-specific IgG4 and IgE were not detected. In addition, serum samples from all participants were investigated for the content of total IgE, using the Human IgE Ready-SET-Go! ELISA (eBioscience) according to the manufacturer’s instructions. Plates were then read at 450 nm as mentioned above.

### Statistical analysis

Statistical analyses were performed using the software SPSS (IBM SPSS Statistics 22; Armonk, NY) and GraphPad PRISM version 5.02 for Windows (GraphPad Software, Inc., La Jolla, USA, www.graphpad.com). *P* values of less than 0.05 were considered statistically significant. Since most of the variables were not normally distributed, the following tests were performed: Kruskal-Wallis-test was performed to compare three groups, followed by a Mann-Whitney–U tests for further pairwise comparison of the group. For comparisons of continuous parameters the Spearman correlation was used. To assess differences between non immunological parameters the Chi-square test was used. To determine potential indicators for the different infections statuses, immune and epidemiological data were also assessed using two sets of binary logistic regression models. For both analyses, age was subgrouped into "young" (4–9 years old), "adolescent" (10–19 years old) and "adult" (20–80 years old) and the covariates "exposure" and "non exposure" refer to individuals obtaining daily water from the canal region or delivered by donkey carts or pipes. In the first analysis the covariates age and exposure were assessed with each individual cytokine or immunoglobulin. Hence, no intercomparison between cytokines was performed (Table 2). In a second multivariate analysis, all epidemiological (exposure, latrine, education, age (subgrouped), co-infection, gender) and immune parameters were added as covariates and compared with one another (Table 3). Comparisons were made between "infected and non-infected", "egg^+^ vs *Sm* uninf", "*Sm*PCR^+^ vs *Sm* uninf" and "*Sm*PCR^+^ vs egg^+^". The second analysis was performed as a stepwise logistic regression with forward selection using thresholds of *P*<0.1 for entry of variables into the model. The odds ratio (OR), 95% confidence intervals (CI) and p values were used as estimates of the effect of each variable [21].

**Table 2:** Summary of binary multivariable regression analysis between the different patient groups: Relation of the covariates age, exposure and individual cytokines or Igs.

	Covariate	OR	CI	p value	Age	Exposure
**Infected vs.uninfected**						
	IL-1β (pg/ml)	0.999	[0.996–1.001]	0.380		
	IL-2 (pg/ml)	0.998	[0.996–1.001]	0.239		
	IL-4 (pg/ml)	0.999	[0.989–1.010]	0.882		
	**IL-5 (pg/ml)**	**0.996**	**[0.993–1.000]**	**0.039**	**Yes (1+2)**	**Yes**
	IL-6 (pg/ml)	1.000	[0.999–1.001]	0.505		
	IL-8 (ng/ml)	1.006	[0.956–1.059]	0.807		
	IL-9 (pg/ml)	0.988	[0.973–1.004]	0.137		
	IL-10 (pg/ml)	0.997	[0.982–1.012]	0.671		
	IL-12p70 (pg/ml)	0.994	[0.983–1.004]	0.227		
	IL-13 (pg/ml)	1.005	[0.992–1.019]	0.418		
	IL-17A (pg/ml)	0.997	[0.993–1.002]	0.275		
	IL-22 (pg/ml)	0.998	[0.996–1.001]	0.132		
	IFN-γ (pg/ml)	0.999	[0.990–1.008]	0.790		
	TNF-α (pg/ml)	0.998	[0.993–1.004]	0.512		
	**total IgE (ng/ml)**	**1.158**	**[1.040–1.289]**	**0.007**	**Yes (1+2)**	**Yes**
	**SEA IgG4 (OD)**	**16.021**	**[6.509–39.431]**	**0.000**	**Yes (1+2)**	**Yes**
	SEA IgE (OD)	0.763	[0.223–2,603]	0.665		
**egg**^**+**^ **vs *Sm* uninf**						
	IL-1β (pg/ml)	1.000	[0.997–1.002]	0.776		
	IL-2 (pg/ml)	0.999	[0.997–1.002]	0.560		
	IL-4 (pg/ml)	0.997	[0.985–1.009]	0.588		
	**IL-5 (pg/ml)**	**0.996**	**[0.991–1.000]**	**0.054**	**Yes (1+2)**	**Yes**
	IL-6 (pg/ml)	1.000	[0.999–1.002]	0.458		
	IL-8 (ng/ml)	1.004	[0.952–1.058]	0.886		
	IL-9 (pg/ml)	0.969	[0.931–1.008]	0.117		
	IL-10 (pg/ml)	0.996	[0.979–1.013]	0.616		
	**IL-12p70 (pg/ml)**	**0.978**	**[0.958–0.997]**	**0.027**	**Yes (1+2)**	**Yes**
	IL-13 (pg/ml)	1.010	[0.995–1.024]	0.201		
	IL-17A (pg/ml)	0.996	[0.997–1.002]	0.201		
	IL-22 (pg/ml)	0.998	[0.995–1.000]	0.109		
	IFN-γ (pg/ml)	0.998	[0.987–1.008]	0.651		
	TNF-α (pg/ml)	0.997	[0.990–1.004]	0.365		
	**total IgE (ng/ml)**	**1.260**	**[1.104–1.437]**	**0.001**	**Yes (1+2)**	**Yes**
	**SEA IgG4 (OD)**	**13.644**	**[5.228–34.872]**	**0.000**	**Yes (1)**	**Yes**
	SEA IgE (OD)	0.983	[0.237–4.082]	0.981		
***Sm*PCR**^**+**^ **vs *Sm* uninf**						
	**IL-1β (pg/ml)**	**0.995**	**[0.991–1.000]**	**0.036**	**Yes (1+2)**	**No**
	**IL-2 (pg/ml)**	**0.993**	**[0.986–1.000]**	**0.062**	**Yes (1+2)**	**No**
	IL-4 (pg/ml)	1.000	[0.988–1.013]	0.958		
	IL-5 (pg/ml)	0.997	[0.993–1.002]	0.220		
	IL-6 (pg/ml)	1.000	[0.999–1.001]	0.570		
	IL-8 (ng/ml)	1.023	[0.955–1.097]	0.513		
	IL-9 (pg/ml)	0.997	[0.981–1.013]	0.679		
	IL-10 (pg/ml)	0.995	[0.975–1.015]	0.606		
	IL-12p70 (pg/ml)	0.998	[0.987–1.009]	0.739		
	IL-13 (pg/ml)	0.996	[0.981–1.011]	0.603		
	IL-17A (pg/ml)	0.998	[0.992–1.004]	0.533		
	IL-22 (pg/ml)	0.999	[0.996–1.002]	0.558		
	IFN-γ (pg/ml)	0.999	[0.987–1.010]	0.802		
	TNF-α (pg/ml)	0.999	[0.992–1.006]	0.771		
	total IgE (ng/ml)	1.076	[0.952–1.217]	0.240		
	**SEA IgG4 (OD)**	**35.396**	**[9.282–134.984]**	**0.000**	**Yes (1+2)**	**No**
	SEA IgE (OD)	0.575	[0.122–2.719]	0.485		
**egg**^**+**^ **vs *Sm*PCR** ^**+**^						
	**IL-1β (pg/ml)**	**1.005**	**[1.001–1.009]**	**0.024**	**No**	**Yes**
	**IL-2 (pg/ml)**	**1.007**	**[1.001–1.013]**	**0.026**	**No**	**Yes**
	IL-4 (pg/ml)	1.002	[0.986–1.017]	0.834		
	IL-5 (pg/ml)	0.999	[0.992–1.005]	0.739		
	IL-6 (pg/ml)	1.000	[0.999–1.001]	0.793		
	IL-8 (ng/ml)	0.980	[0.927–1.035]	0.475		
	IL-9 (pg/ml)	0.978	[0.946–1.011]	0.196		
	IL-10 (pg/ml)	1.010	[0.989–1.030]	0.366		
	IL-12p70 (pg/ml)	0.991	[0.975–1.006]	0.230		
	**IL-13 (pg/ml)**	**1.017**	**[1.000–1.034]**	**0.051**	**No**	**Yes**
	IL-17A (pg/ml)	0.998	[0.992–1.005]	0.642		
	IL-22 (pg/ml)	0.998	[0.996–1.001]	0.195		
	IFN-γ (pg/ml)	1.003	[0.991–1.016]	0.596		
	TNF-α (pg/ml)	1.001	[0.992–1.010]	0.813		
	**total IgE (ng/ml)**	**1.103**	**[0.992–1.228]**	**0.070**	**No**	**Yes**
	SEA IgG4 (OD)	1.788	[0.779–4.115]	0.170		
	SEA IgE (OD)	2.314	[0.521–10.30]	0.270		

Data were assessed using a binary multivariable regression analysis using the covariates age and exposure with each individual cytokine or immunoglobulin. The age covariate was subgrouped into "young" (4–9 years old), "adolescent" (10–19 years old) and "adult" (20–80 years old). The covariate "exposure" referred to the daily water source and therefore "exposed" means individuals obtained water from the canal region whereas "not exposed" means water sources were donkey carts or pipes. Analysis was performed between the three different groups and the results depict significant parameters with OR (odds ratio), 95% CI and p values. When the OR is above 1 then associations are with the group which is listed first. The columns age and exposure state whether or not there was an association with the cytokines and immunoglobulins that were significant between the groups. Group 1 refers to "young" and group 2 refers to "adolescent"; no associations with "adult" were found.

**Table 3:** Summary of binary multivariable regression analysis between the different patient groups: relation of the covariates cytokines, immunoglobulins, age and epidemiological parameters.

	Covariate	OR	CI	p value
**infected versus uninfected**				
	SEA IgG4 (OD)	19.384	[7.967–47.161]	0.000
	IL-5 (pg/ml)	0.994	[0.990–0.998]	0.005
	not exposed	0.368	[0.162–0.838]	0.017
	age group			0.025
	young	5.230	[1.472–18.584]	0.011
	adolescent	3.779	[1.222–11.694]	0.021
	IL-12p70 (pg/ml)	0.990	[0.979–1.002]	0.090
**egg**^**+**^ **versus *Sm* uninf**				
	SEA IgG4 (OD)	19.784	[7.718–50.530]	0.000
	IL-5 (pg/ml)	0.993	[0.987–0.999]	0.015
	no latrine in house	5.376	[1.990–14.518]	0.001
	age group			0.003
	young	16.020	[3.183–80.633]	0.001
	adolescent	4.300	[1.188–15.562]	0.026
	IL-13 (pg/ml)	1.030	[1.009–1.052]	0.005
	IL-12p70 (pg/ml)	0.963	[0.938–0.989]	0.006
	IL-8 (ng/ml)	1.099	[1.004–1.203]	0.040
	IgE (ng/ml)	1.201	[0.983–1.469]	0.073
	IL-10 (pg/ml)	1.042	[0.991–1.095]	0.110
***Sm*PCR**^**+**^ **versus *Sm* uninf**				
	SEA IgG4 (OD)	26.205	[7.529–91.210]	0.000
	IL-5 (pg/ml)	0.995	[0.991–1.000]	0.034
	female	0.404	[0.145–1.128]	0.083
**egg**^**+**^ **versus *Sm*PCR**^**+**^				
	no latrine in house	6.211	[2.915–13.157]	0.000
	not coinfected	0.241	[0.106–0.547]	0.001
	IgE (ng/ml)	1.160	[1.016–1.324]	0.028
	not exposed	5.780	[1.077–31.250]	0.041
	female	2.293	[0.979–5.376]	0.056
	IL-12p70 (pg/ml)	0.958	[0.931–0.987]	0.004
	IL-10 (pg/ml)	1.033	[0.998–1.068]	0.061
	IL-5 (pg/ml)	0.987	[0.975–0.998]	0.025
	IL-1β (pg/ml)	1.008	[1.001–1.015]	0.035
	IL-2 (pg/ml)	1.008	[1.000–1.017]	0.061

Data were assessed using a binary multivariable regression analysis using the parameters exposure (daily water source), latrine, education, age, co-infection, gender and immune parameters as covariates. The age covariate was further subgrouped into "young" (4–9 years old), "adolescent" (10–19 years old) and "adult" (20–80 years old). Analysis was performed between the three different groups and the results depict significant parameters with OR (odds ratio) and CI values and p values. When the OR is above 1 then associations are with the group which is listed first. Regarding cytokine values, the OR of 0.992 for IL-1β in " egg^+^ vs *Sm*PCR^+^" for example, means that increases in cytokine levels for one unit (pg) signifies an association with the egg^+^ group.

## Results

### Characteristics of the study population

**Fig 2:**
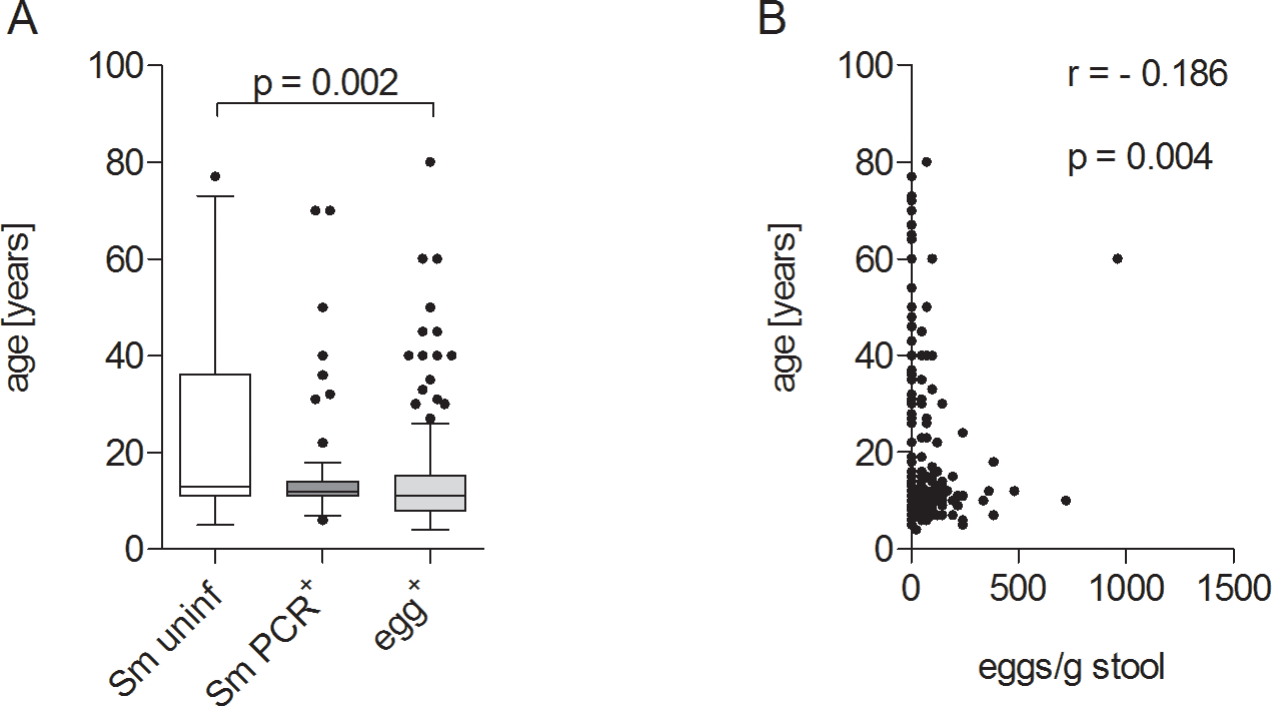
Gender and age distribution of study participants. Study participants were grouped according to absence (*Sm* uninf = 61) or presence of *S*. *mansoni* DNA in blood (*Sm*PCR^+^ = 63) or *S*. *mansoni* eggs in stool samples (egg^+^ = 110). (A) Distribution of age within all three groups. Data shows box whiskers with median, interquartile ranges and outliers. Statistical significances between the indicated groups were obtained after Kruskal-Wallis and Mann-Whitney-U tests. (B) Correlation of age with egg number, statistical significances were tested using the Spearman correlation test.

Within the study population of 234 participants, 110 had *S*. *mansoni* eggs in their stool (egg^+^; median 72 eggs/g of stool, range 24–960 eggs/g of stool), 63 individuals were egg-negative but *S*. *mansoni* PCR-positive (*Sm*PCR^+^), and 61 were negative in both diagnostic parameters (*Sm* uninf). Within the egg^+^ group there was a higher percentage of females when compared to both other groups (p<0.001, Table 1). With regards to age, *Sm* uninf individuals ranged between 5 and 77 years (median 13 years), *Sm*PCR^+^ people from 6 to 70 years (median 12 years), and egg^+^ individuals between 4 and 80 years (median 11 years). Using a Chi square test a significant association between infection state and age was observed (X2: p = 0.003). Comparison of age further revealed that *Sm* uninf individuals were significantly older than egg^+^ patients (Fig 2A) and age was inversely correlated with the number of eggs/g of stool (Fig 2B) which was also shown previously in *S*. *haematobium* infection [22].

### Rates of *S*. *mansoni* infection correlate with water supply and sanitation facilities

Individuals from all three groups were analysed for other parasitic infections as shown in Table 1. Co-infections with *H*. *nana*, *G*. *lamblia* and *E*. *histolytica* were found in all three groups but the highest percentage of co-infections was found in the egg^+^ group, but within this group there were equal numbers of co-infected and *S*. *mansoni* only cases (S1 Table). Using a Chi-square test, we observed a significant association with the *S*. *mansoni* infection status *per se*, thus, egg^+^ participants had more co-infections than *Sm* uninf or *Sm*PCR^+^ individuals (Table 1). The co-infection status of the cohort was also inversely correlated to age (r = - 0.235, p<0.001). Participants were asked about the availability of latrines and their drinking water supply. Very few egg^+^ individuals had latrines in their households (<20%) when compared to the other groups. 90.9% of the egg^+^ participants obtained their drinking water from canals, whilst 9.1% obtained their water from pipes, the only two sources of water recorded for this population. Both parameters were significantly associated with infection status (p<0.001, Table 1). Participants were further asked about their educational background (no schooling, primary school, high school or university). The educational background was also significantly associated with infection status (p<0.001, Table 1) and positively correlated with knowledge about *Schistosoma* infections *per se* (r = 0.210, p = 0.001).

### Egg^+^ individuals have elevated levels of circulating IL-13 but not IL-4 or IL-5

**Fig 3:**
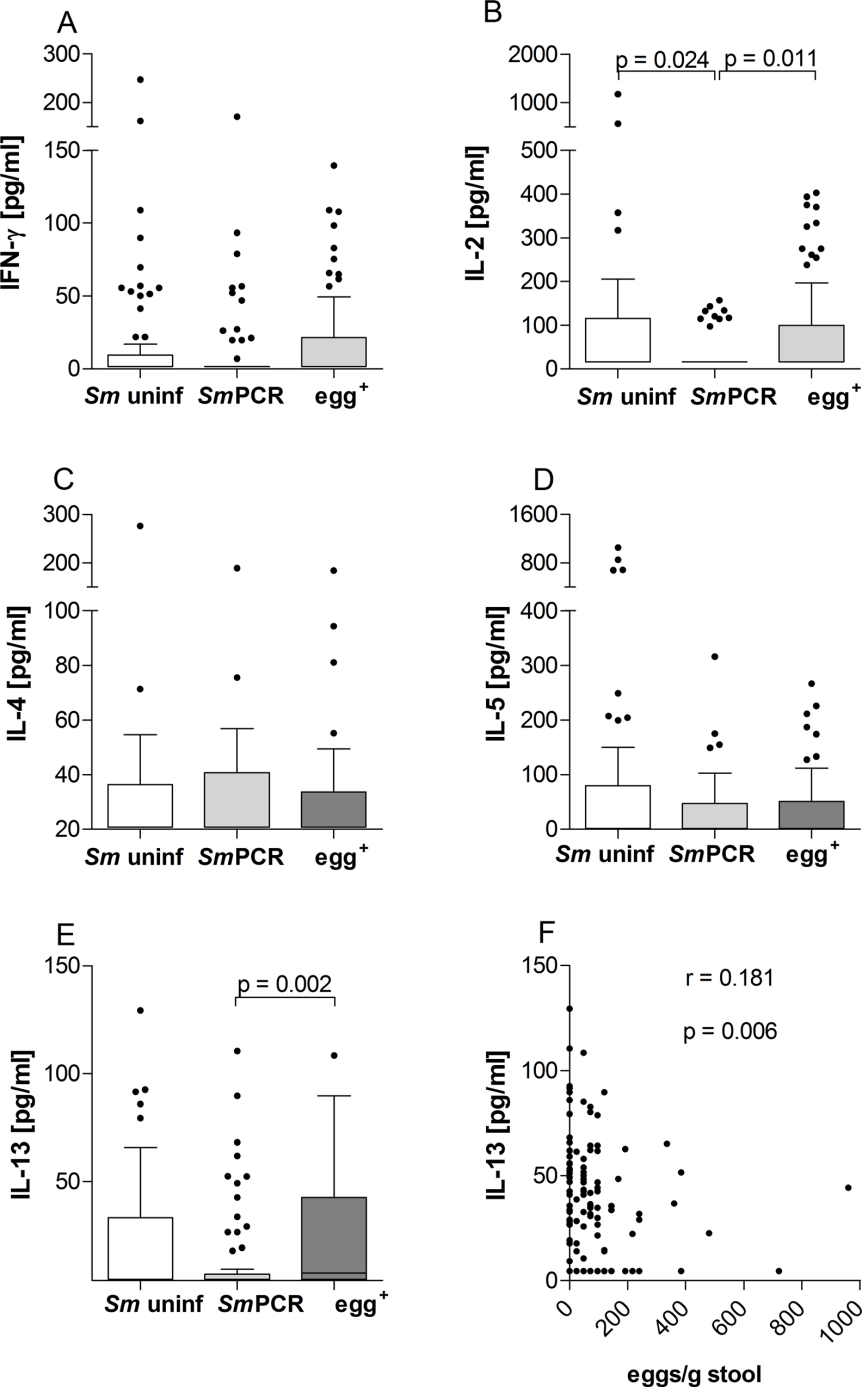
Systemic IL-13 is elevated in patently-infected *S. mansoni* individuals. Serum from participants (n = 234) was analyzed for the production of IFN-γ (A), IL-2 (B), IL-4 (C), IL-5 (D) and IL-13 (E) using FlowCytomix technology. Graphs show box whiskers with median, interquartile ranges and outliers. Statistical significances between the indicated groups were obtained after Kruskal-Wallis and Mann-Whitney-U tests. IL-13 levels were correlated with the number of eggs (F) and tested for statistical significance using the Spearman correlation test.

To survey the cytokine milieu of the different groups, serum samples of all individuals were analysed for Th1 associated cytokines IFN-γ and IL-2 and classical Th2 cytokines IL-4, IL-5 and IL-13 using multiplex kits (Fig 3). With regards to IFN-γ, no significant differences could be observed between the groups (Fig 3A). Levels of IL-2 however, were significantly lower in individuals in the *Sm*PCR^+^ group when compared to both other groups (Fig 3B). Levels of IL-4 and IL-5 were not significantly altered (Fig 3C and 3D). Interestingly, IL-13 levels were also significantly lower in the *Sm*PCR^+^ individuals when compared to those that were egg^+^ group (Fig 3E). Moreover, upon further analysis, whereas IL-13 levels positively correlated with egg load (Fig 3F) IL-2 levels did not.

### Higher levels of IL-6 in patently-infected individuals correlate with egg burden

**Fig 4:**
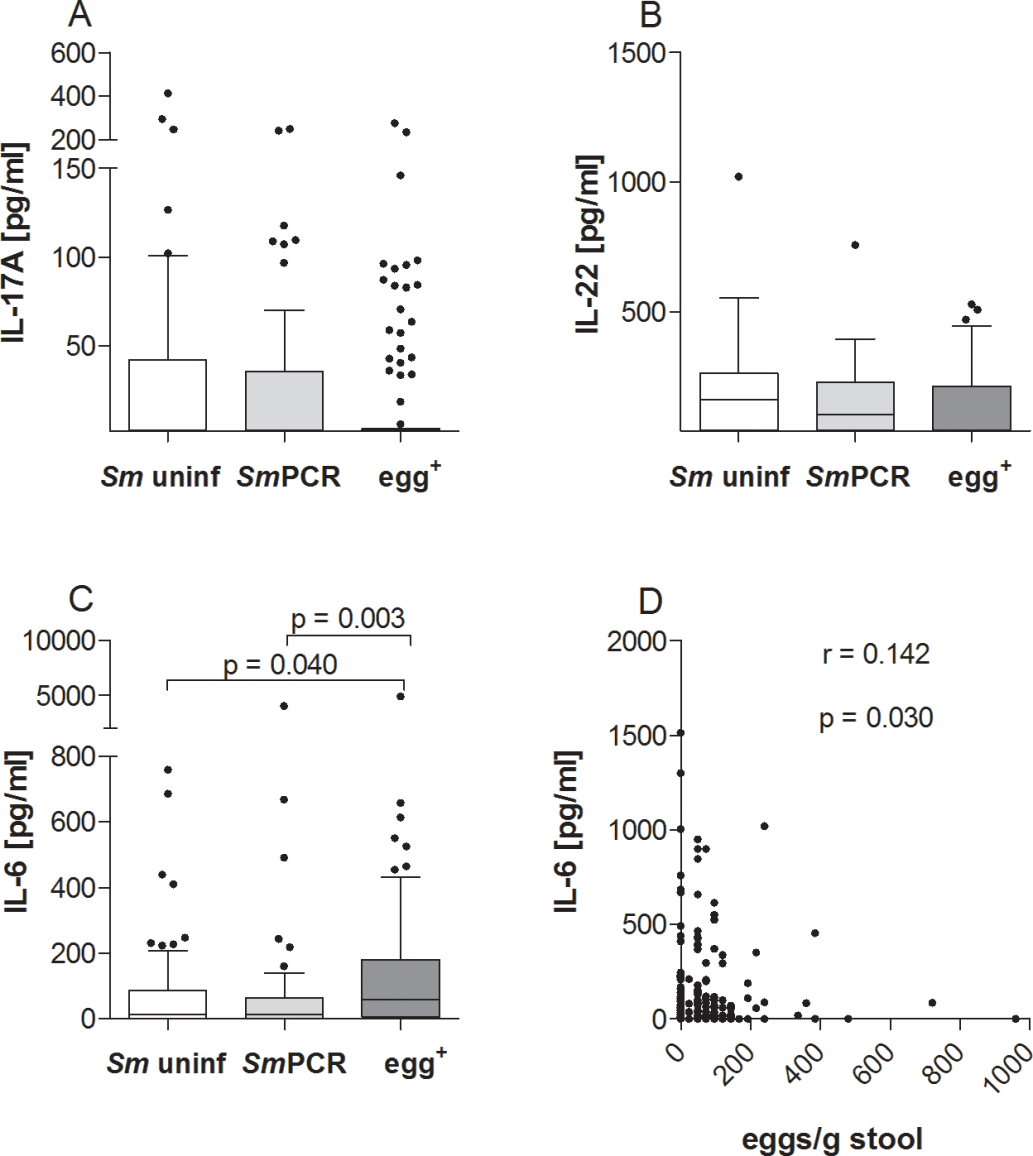
Patent infections of *S. mansoni* elevate IL-6 responses. Serum from all three groups was analyzed for the production of IL-17A (A), IL-22 (B) and IL-6 (C) using FlowCytomix technology. Graphs show box whiskers with median, interquartile ranges and outliers. Statistical significances between the indicated groups were obtained after Kruskal-Wallis and Mann-Whitney-U tests. IL-6 levels were correlated with the number of eggs (D) and tested for statistical significance using the Spearman correlation test.

Since it was previously shown that Th17 cell responses are associated with severe pathology in *S*. *haematobium-*infected individuals [9] we also investigated serum samples for the content of IL-17A and factors related to the induction of Th17 responses, that is IL-22 and IL-6 [23, 24] (Fig 4). With regards to IL-17A and IL-22, there were no significant differences between all three groups (Fig 4A and 4B), but IL-6 was significantly increased in egg^+^ patients when compared to either the *Sm* uninf or *Sm*PCR^+^ groups (Fig 4C). Moreover, the amount of IL-6 significantly correlated with the number of excreted eggs (Fig 4D).

### IL-1β levels are significantly lower in *Sm*PCR^+^ individuals

**Fig 5:**
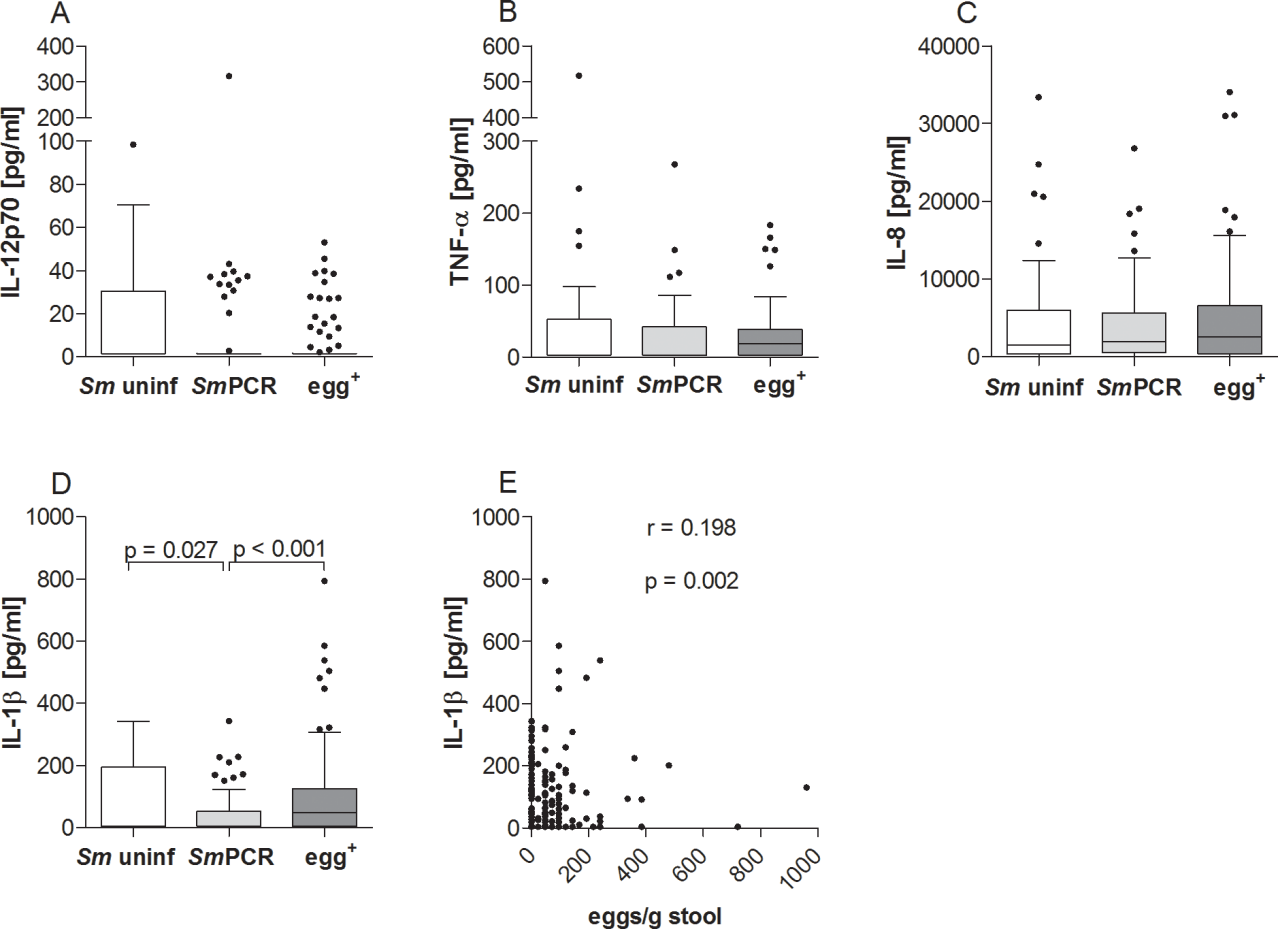
Lower levels of systemic IL-1β in SmPCR^+^ individuals. Individual serum samples from all three groups were analyzed for the production of IL-12p70 (A), TNF-α (B), IL-8 (C) and IL-1β (D) using FlowCytomix technology or ELISA (IL-8). Graphs show box whiskers with median, interquartile ranges and outliers. Statistical significances between the indicated groups were obtained after Kruskal-Wallis and Mann-Whitney-U tests. IL-1β levels were correlated with the number of eggs (E) and tested for statistical significance using the Spearman correlation test.

Next, we measured levels of the innate-related cytokines IL-12p70, TNF-α, IL-1β and IL-8 in the serum samples from study participants. Although levels of IL-12p70, TNF-α, and IL-8 (Fig 5A, 5B and 5C) were not significantly different between the three groups, IL-1β levels were significantly lower in individuals from the *Sm*PCR^+^ group when compared to the other groups (Fig 5D). In addition, the number of eggs/g stool also significantly correlated to levels of IL-1β (Fig 5E) but not to the other cytokines.

### Opposing levels of IL-9 and IL-10 in patently-infected *S*. *mansoni* individuals

**Fig 6:**
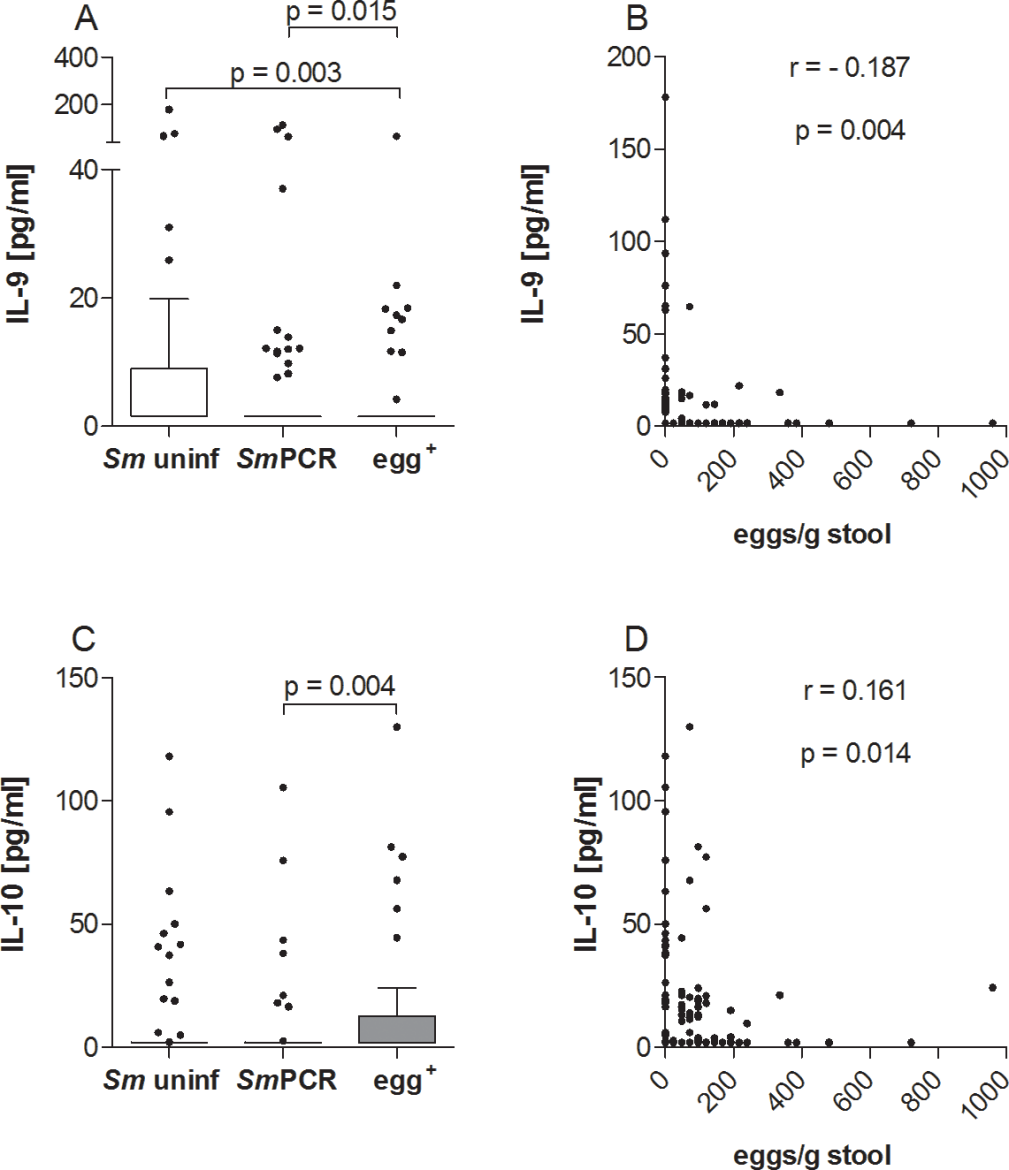
Systemic levels of IL-10 or IL-9 showed opposing profiles in patently infected individuals. Serum from all three groups was analyzed for the production of IL-9 (A) and IL-10 (C) using FlowCytomix technology. Graphs show box whiskers with median, interquartile ranges and outliers. Statistical significances between the indicated groups were obtained after Kruskal-Wallis and Mann-Whitney-U tests. Levels of IL-9 (B) and IL-10 (D) were correlated with the number of eggs and tested for statistical significance using the Spearman correlation test.

Besides Th1, Th2 and innate cytokines we also measured IL-10 and IL-9 from the serum samples of all study participants. With regards to IL-9 we found significantly lower levels in serum samples in the egg^+^ group when compared to both other groups (Fig 6A), which was further emphasized by a negative correlation with the egg burden (Fig 6B). Note, IL-9 levels were very low and only few individuals had detectable levels of this cytokine. In contrast, levels of IL-10 were significantly higher in the egg^+^ group when compared to the *Sm*PCR^+^ group (Fig 6C) and significantly correlated to the number of eggs in stool samples (Fig 6D).

### Increased amounts of total IgE and SEA-specific IgG4 in infected individuals

**Fig 7:**
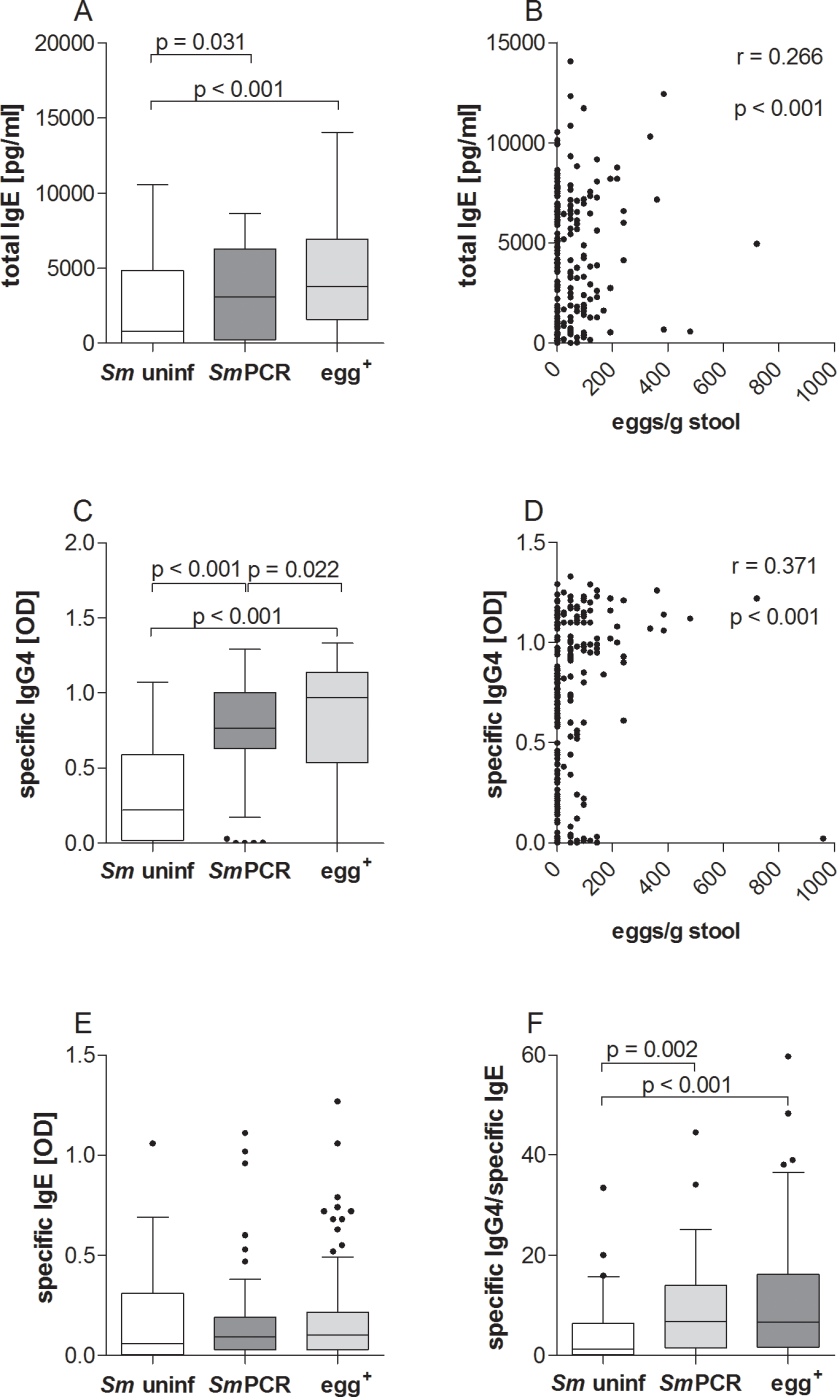
Higher levels of SEA-specific IgG4 but not IgE production in infected individuals. Serum from all three groups was analyzed for the concentration of total IgE (A) and the optical density of SEA-specific IgG4 (C) and IgE (E) using ELISA. (F) depicts the ratio of antigen-specific IgG4/IgE. Graphs show box whiskers with median, interquartile ranges and outliers. Statistical significances between the indicated groups were obtained after Kruskal-Wallis and Mann-Whitney-U tests. Levels of total IgE and SEA-specific IgG4 immunoglobulins were further correlated with egg load (B and D respectively) and tested for statistical significance using the Spearman correlation test.

Since immunoglobulins are relevant for the outcome of an infection, we measured SEA-specific IgG4 and IgE antibody levels and also total IgE as depicted in Fig 7. Total IgE was significantly increased in both infected groups when compared to the *Sm* uninf individuals and additionally, was higher in egg^+^ patients when compared to the *Sm*PCR^+^ individuals (Fig 7A). Moreover, the number of eggs correlated significantly with levels of total IgE (Fig 7B). SEA-specific IgG4 levels were significantly increased in both infected groups when compared to *Sm* uninf individuals (Fig 7C) and also correlated with egg load (Fig 7D). In addition, SEA-specific IgG4 was elevated in the egg^+^ group in comparison to the *Sm*PCR^+^ individuals. However, SEA-specific IgE was not significantly altered between the groups (Fig 7E), but the ratio of SEA-specific IgG4/IgE was significantly increased in both infected groups compared to *Sm* uninf individuals (Fig 7F).

### Multivariable regression analysis indicates that *S*. *mansoni* infection was strongly associated with SEA-specific IgG4 and *Sm*PCR^+^ individuals are more likely to have reduced peripheral levels of IL-10, IL-1β and IL-2

To gain further insight into potential markers of infection, we performed a more detailed statistical analysis using two binary multivariable logistic regression analyses. For both analyses, age was subgrouped into "young" (4–9 years old), "adolescent" (10–19 years old) and "adult" (20–80 years old). In the first analysis we used the covariates age and “exposure” with each individual cytokine or immunoglobulin. Hence, no intercomparison between cytokines was performed. The covariate "exposure" referred to water obtained from the canal region whereas "not exposed" meant water was delivered by donkey cart or pipes. Table 2 shows the outcome of this analysis between "infected vs uninfected", "egg^+^ vs *Sm* uninf", "*Sm*PCR^+^ vs *Sm* uninf" and "egg^+^ vs *Sm*PCR^+^". Interestingly, SEA-specific IgG4 was highly associated with infection since this parameter was significant in all comparisons except "egg^+^ vs *Sm*PCR^+^". Total IgE levels however were associated with all comparisons except "*Sm*PCR^+^ vs *Sm* uninf". With regards to cytokines, IL-5 was interestingly associated with no schistosome infection and was associated with both age and exposure. The cytokines IL-1β and IL-2 on the other hand were more associated with non-egg schistosome infected individuals since these parameters were significant in "egg^+^ vs *Sm*PCR^+^" and "*Sm*PCR^+^ vs *Sm* uninf". These data confirm the outcome of the cytokine measurements shown in Figs 3B and 5D.

In the second analysis, all epidemiological (exposure, latrine, education, age, co-infection, gender) and immune parameters were added as covariates and compared with one another. Table 3 shows the outcome of this analysis, again comparing the 4 different infection combinations. With regards to age, our analysis revealed that infection and an egg^+^ status was linked to young and adolescent groups. SEA-specific IgG4 was revealed to be the most highly associated factor with infection. Both significance and odds-ratio (OR) were high when comparing "infected vs non-infected", "egg^+^ vs *Sm* uninf" and "*Sm*PCR^+^ vs *Sm* uninf" groups. This was confirmed upon comparison between " egg^+^ vs *Sm*PCR^+^" since this covariate was not significant indicating that between the two infected groups this parameter was comparable. As observed in the primary regression analysis, systemic IL-5 levels were associated with uninfected groups as was IL-12p70 (infected vs uninfected and egg^+^ vs *Sm* uninf). When comparing egg^+^ and *Sm*PCR^+^ groups, co-infection was revealed as a dominant epidemiological factor and with regards to cytokines, IL-12p70, IL-10, IL-1β and IL-2 were also significantly associated. Thus, the combined multivariate analysis revealed that egg^+^ *S*. *mansoni* infection was strongly associated with SEA-specific IgG4 whereas non-egg^+^ but *Sm*PCR^+^ individuals were more likely to have reduced peripheral levels of IL-10, IL-1β and IL-2, which actually confirms the data shown in Figs 6C, 5D and 3B respectively.

## Discussion

The diverse outcomes of schistosome infections are determined by the balance of different immune responses directed against larval and adult stages of the parasite, as well as parasite eggs trapped in the tissues [11]. In this retrospective study our aim was to distinguish potential biomarkers or characteristics of schistosome infection using a cohort from endemic areas in the Sudan. Thus, we compared the immune profiles of non-infected and *S*. *mansoni*-infected individuals that were a) positive for eggs in stool or b) egg-negative but *Sm*PCR^+^ for worm DNA in sera. Our cohort spanned a large geographical area and included individuals that had had varying degrees of contact to infectious water sources and older persons that may have developed a level of resistance through previous exposure. Since such epidemiological factors and age are considered to shape immune responses we performed two binary multivariable logistic regression analyses to determine potential factors associated with infection. Since we had a large age range in our cohort we subgrouped our individuals into young (4–9 years old), adolescent (10–19 years old) and adult (>20 years old). In endemic areas, initial infections by schistosomes can occur in very young children (around 2 years) and parasite burdens increase in intensity during the following 10 years as new worms colonise the child’s body [22]. In adolescence, infections decline and since this trend also occurs in highly endemic fishing communities it is considered that a type of immunity arises in older years that hinders re-infection [25, 26]. This supports other similar findings that the lower prevalence of infection in older individuals results from the development of anti-parasite immunity rather than reduced contact with infectious water sources [3]. Deterministic models dealing with different levels of infection, revealed that infection intensity peaked at an earlier age in areas with higher levels of infection transmission rates when compared to areas with lower transmission rates [22, 27]. In our second regression analysis both young and adolescent age categories were found to be significantly associated with infection and moreover with egg^+^ individuals. Age was not revealed to be a significant factor when comparing egg^+^ and *Sm*PCR^+^ individuals. In accordance with these findings, our study revealed a negative correlation between eggs/g stool and age (Fig 2C). Interestingly, *in vivo* studies using laboratory mice revealed that although aging did not affect parasite load, young mice (2 months) developed larger granulomas in the acute phase of schistosome infection: a phenomenon not observed in older mice (18 months). The authors also showed that young infected mice produced increased amounts of IFN-γ, IL-4 and IL-13 whereas older mice already had higher levels of IL-4 and IL-10 *per se* [28]. In the study described here, IFN-γ and IL-4 levels were not significantly altered between the groups (Fig 3A and 3C) and these cytokines were not associated in either of the regression analyses either. These findings correlate with the systemic immune profiles previously observed in *S*. *haematobium*-infected patients [6] indicating that they are not good markers of schistosome infection. IL-13 levels were however significantly lower in *Sm*PCR^+^ individuals and were significantly associated with egg^+^ individuals in the primary regression analysis comparing egg^+^ and *Sm*PCR^+^ groups (p 0.051). IL-13 is a profibrotic agent and its association with egg-induced immunopathology during schistosomiasis is well documented [29]. Caldas *et al*., however, reported that levels of this cytokine were not related to the intensity of infection and concluded that although IL-13 was involved in the development of severe fibrosis it did not trigger the initial process [7]. Our current findings strongly indicate that low peripheral IL-13 levels are a marker of non-patent or low egg intensity infections a facet that has not been observed in *S*. *haematobium*-infected individuals [6].

Following our second regression analysis, egg^+^ individuals are also more likely to have poor hygiene facilities at home since the covariate "no latrine in house" was significantly associated in both "egg^+^ vs *Sm* uninf" and "egg^+^ vs *Sm*PCR^+^" comparisons. The covariate "not exposed" was also significantly associated when comparing "infected vs uninfected". These data confirmed our initial assessment that there was an association between lower infection status and better access to proper latrines and clean drinking water. Females were also more likely to be egg^+^ but this is probably due to domestic and occupational contact with fresh water sources. In agreement with these findings, a previous epidemiological study from the Sudan showed that among adults, ongoing *S*. *haematobium* infections were associated with refusal to use available latrine facilities and used snail contaminated water sources for daily household purposes such as washing [30]. Another study by Lee *et al*. also confirmed the importance of clean water supplies for the control of urogenital schistosomiasis in the Sudan [31]. However, the most highly associated factor with infection, for both egg^+^ and *Sm*PCR^+^ groups was the presence of SEA-specific IgG4. Levels of SEA-specific IgG4 also correlated positively with the number of eggs/g stool. Moreover, whereas SEA-specific IgE levels were not significantly different between the three groups, total IgE levels were higher in the patently-infected group and also correlated with egg burden. Total IgE was also significantly associated with the egg^+^ group in the regression analysis when compared to both uninfected and *Sm*PCR^+^ cohorts. Research in endemic areas of schistosomiasis has revealed that schistosome adult worm antigen (SWA) specific IgE levels increased with age and correlated with immunity to infection [32–35]. Interestingly, multiple rounds of praziquantel treatment appears to break this resistance [36]. IL-5 responsiveness is also considered a key component in immunity since plasma levels spike after treatment (*S*. *mansoni* and *S*. *haematobium*) and were dependent on SWA-specific IgE levels, eosinophil numbers and infection intensity [26, 37, 38]. In our study, peripheral IL-5 levels were comparable between the groups but in both regression analyses this cytokine was associated with the uninfected groups. Of interest, a recent comparisons of urogenital and intestinal schistosomiasis concluded that IL-5 responses to *S*. *haematobium* infections were stronger than those elicited by *S*. *mansoni* [10]. In this study we were unable to measure SWA-specific IgG4 or IgE but this parameter should be addressed in future studies especially since Negrao-Correa and colleagues suggested that worm-specific antibody profiles were good correlates for determining disease severity (pathology) in schistosome-infected individuals and could be used as biomarkers [39]. However, the same group showed no association between both SEA and SWA-specific IgG or IgE antibodies and parasite burden [39].

Balanced immune responses during helminth infections in man are associated with increased regulatory cell types, high IgG4 and elevated IL-10 levels and the latter is thought to control morbidity in human schistosomiasis [40–43]. Lack of IL-10 in mice is detrimental during *S*. *mansoni* infection [44], and depletion of regulatory T cells results in both exaggerated pathology and uncontrolled immune responses [13, 45]. Recently, Scheer *et al*. determined that lymph node and splenic CD4^+^ T cell subsets were the highest producers of IL-10 during chronic schistosomiasis [46]. In our study cohort, peripheral IL-10 levels were higher in egg^+^ individuals when compared to either *Sm* uninf or *Sm*PCR^+^ groups. This was confirmed in the second regression analysis since IL-10 was highly associated with egg^+^ individuals in the "egg^+^ vs *Sm* uninf" and "egg^+^ vs *Sm*PCR^+^" groups. The regression analysis also revealed potential infection associated markers that were not initially apparent. For example, IL-8 levels were significantly associated with egg^+^ individuals. A study by Turner *et al*. [47] showed that in contrast to levels of IL-10, those of TNF-α and IL-8 were not different between uninfected and *S*. *mansoni*-infected individuals following stimulation of whole blood cultures with cercarial excretory/secretory products. A previous study similarly showed that there was no difference between IL-8 levels in plasma samples from *S*. *mansoni* patients and healthy control individuals [48]. Thus although our initial appraisal of the data are in line with these results the regression analysis revealed deeper associations regarding IL-8 that warrant further investigation.

A surprising finding in this study was the decreased level of systemic IL-1β and IL-2 in the *Sm*PCR^+^ cohort compared to *Sm* uninf and those with patent infection. Egg burden also positively correlated to levels of IL-1β and these findings were further substantiated in the regression analysis. Our earlier studies showed that SEA-derived components drive IL-1β production in a NLRP3 inflammasome-dependent manner, and thus influences immune responses and granuloma formation (or immunopathology) [49]. Alongside IL-6, this pro-inflammatory cytokine is also required for the generation of Th17 cells [23, 50]. In *S*. *haematobium*-infected children, the frequency of Th17 cells was significantly higher in individuals with bladder pathology compared to infected subjects without pathology and age-matched uninfected individuals [9]. In the present study we did not detect any significant differences with regards to systemic IL-17A levels between the groups but it has to be noted that our participants were not considered to be suffering from severe pathology. In addition, IL-22, which is part of the Th17 family [24], was also not significantly different between the three groups and no associations were revealed in the regression analysis either. Levels of IL-6 on the other hand, were increased in sera from egg^+^ individuals which correlated significantly with the egg burden but this factor was not found to be significantly associated in the regression analyses. IL-6 can up-regulate IL-10 production upon exposure to *S*. *mansoni* eggs *in vivo* and the two cytokines together negatively regulate IL-12 and IFN-γ production during the early phase of infection, indicating an anti-inflammatory role of IL-6 during schistosomiasis [51]. Such data support our observed increases in systemic IL-6 and IL-10 in patently-infected individuals and argues for some kind of suppressive milieu.

Surprisingly, although our initial data showed an association between infection status and the level of schooling, this "education" covariate was not a significant parameter in the regression analysis. Nevertheless, egg^+^ individuals were strongly associated with reduced latrine facilities and had higher contact with potentially infected water sources (90.9% of the cohort obtained water from the canal). This indicates that additional health education is required to minimise water contact and thus potential infection and should include information regarding other infections too since this cohort was highly associated with other parasitic infections. Collectively, the study revealed that schistosomiasis remains an important public health problem in the Sudan with a high number of patent individuals. In addition, our new *Sm*PCR revealed another cohort of infected individuals which constituted nearly half of the egg-negative sampled population and provides an avenue for future studies on non-patent infection states. Our study did have several limitations: neither malaria nor associated parameters such as anaemia were diagnosed, only one urine and stool sample was analysed, no malacological surveys were included which would have investigated the population dynamics of the snail intermediate hosts and the relatively small number of the surveyed localities may have negatively influenced the sampled population. In correlation, since no data on snail host populations or cercarial shedding rates were available we have had to assume that these aspects were comparable between the villages. With regards to immune profiles, patent infections of *S*. *mansoni* were strongly associated with young and adolescent cohorts, elevated SEA-specific IgG4, and systemic IL-6, IL-10 and IL-13. Non-egg or low egg intensity infections (*Sm*PCR^+^) on the other hand revealed a unique profile of elevated SEA-specific IgG4 with decreased systemic IL-2 and IL-1β. Further studies should concentrate on deciphering the pathways/mechanisms of these candidate biomarkers of disease which may facilitate the diagnosis of early pathological stages which could help prevent further damage and morbidity.

## Supporting Information

S1 Checklist Strobe checklist. This study adheres to the Strobe guidelines for observational studies.
(DOC)

S1 Fig Distribution of participants in different villages. Study participants were grouped according to their villages which are located in the New Halfa Area (Kassala state) and along the White Nile in Khartoum state.
(TIF)

S1 Table Prevalence of further parasitic infections. Number of participants within each group presenting further parasitic infections. N.B. Some participants were positive for more than one tested parasite.
(DOCX)
