# Supplementary material for: Multivariable Regression Analysis in Schistosoma mansoni-Infected Individuals in the Sudan Reveals Unique Immunoepidemiological Profiles in Uninfected, egg+ and Non-egg+ Infected Individuals
Source: PLoS Negl Trop Dis. 2016 May 6;10(5):e0004629. doi: 10.1371/journal.pntd.0004629 (PMC4859533; doi:10.1371/journal.pntd.0004629)
Supplement: S1 Checklist — This study adheres to the Strobe guidelines for observational studies. (DOC) [file pntd.0004629.s001.doc]

STROBE Statement—Checklist of items that should be included in reports of ***cross-sectional studies***

|  | Item No | Recommendation |
| --- | --- | --- |
| **Title and abstract** | 1 | (*a*) Indicate the study’s design with a commonly used term in the title or the abstract  **This retrospective study evaluated immunoepidemiological aspects in 234 individuals (range 4-85 years old) from Kassala and Khartoum states in 2011.** |
|  |  | (*b*) Provide in the abstract an informative and balanced summary of what was done and what was found.  **Schistosomiasis remains an important public health problem in the Sudan with a high number of patent individuals. In addition, SmPCR diagnostics revealed another cohort of infected individuals with a unique immunological profile and provides an avenue for future studies on non-patent infection states. Future studies should investigate the downstream signalling pathways/mechanisms of IL-2 and IL-1β as potential diagnostic markers in order to distinguish patent from non-patent individuals.** |
| Introduction | | |
| Background/rationale | 2 | Explain the scientific background and rationale for the investigation being reported  **This retrospective study aimed at identifying potential markers of schistosome infection.** |
| Objectives | 3 | State specific objectives, including any prespecified hypotheses  **Pages 5-6** |
| Methods | | |
| Study design | 4 | Present key elements of study design early in the paper  **Page 5-6** |
| Setting | 5 | Describe the setting, locations, and relevant dates, including periods of recruitment, exposure, follow-up, and data collection  **Page 7, Figure 1.** |
| Participants | 6 | (*a*) Give the eligibility criteria, and the sources and methods of selection of participants  **Page 7.** |
| Variables | 7 | Clearly define all outcomes, exposures, predictors, potential confounders, and effect modifiers. Give diagnostic criteria, if applicable  **Page 7/8.** |
| Data sources/ measurement | 8* | For each variable of interest, give sources of data and details of methods of assessment (measurement). Describe comparability of assessment methods if there is more than one group  **Page 7-12 (Methods).** |
| Bias | 9 | Describe any efforts to address potential sources of bias  **n/a** |
| Study size | 10 | Explain how the study size was arrived at  **Between March and October 2011, 770 individuals participated in this Ministry of Health survey and the distribution of this sampling is shown in S1. Of those 770 individuals 110 were S. mansoni egg positive in stool samples. In this retrospective study, we assessed a further 124 individuals that were age, gender and village matched via S. mansoni PCR. Those individuals were then subdivided into S. mansoni uninfected (Sm uninf, n=61) and SmPCR+ egg-negative (n=63). Table 1 shows details about the infection groups within the study cohort.** |
| Quantitative variables | 11 | Explain how quantitative variables were handled in the analyses. If applicable, describe which groupings were chosen and why  **Statistical analyses were performed using the software SPSS (IBM SPSS Statistics 22; Armonk, NY) and GraphPad PRISM version 5.02 for Windows (GraphPad Software, Inc., La Jolla, USA, www.graphpad.com). P values of less than 0.05 were considered statistically significant. Since most of the variables were not normally distribution, the following tests were performed: Kruskal-Wallis-test was performed to compare three groups, followed by a Mann-Whitney–U tests for further pairwise comparison of the group. For comparisons of continuous parameters the Spearman correlation was used. To assess differences between non immunological parameters the Chi-square test was used.** |
| Statistical methods | 12 | (*a*) Describe all statistical methods, including those used to control for confounding  **Page 11-12** |
|  |  | (*b*) Describe any methods used to examine subgroups and interactions  **Page 11-12** |
|  |  | (*c*) Explain how missing data were addressed  **No missing data** |
|  |  | (*d*) If applicable, describe analytical methods taking account of sampling strategy  **Page 8, 9-10** |
|  |  | (*e*) Describe any sensitivity analyses  **Page 9-10 - PCR establishment** |
| Results | | |
| Participants | 13* | (a) Report numbers of individuals at each stage of study—eg numbers potentially eligible, examined for eligibility, confirmed eligible, included in the study, completing follow-up, and analysed  **Page 7** |
|  |  | (b) Give reasons for non-participation at each stage  **n/a** |
|  |  | (c) Consider use of a flow diagram  **n/a** |
| Descriptive data | 14* | (a) Give characteristics of study participants (eg demographic, clinical, social) and information on exposures and potential confounders  **Page 7** |
|  |  | (b) Indicate number of participants with missing data for each variable of interest  **n/a** |
| Outcome data | 15* | Report numbers of outcome events or summary measures  **Pages 13-20 Figure 2-7** |
| Main results | 16 | (*a*) Give unadjusted estimates and, if applicable, confounder-adjusted estimates and their precision (eg, 95% confidence interval). Make clear which confounders were adjusted for and why they were included  **Provided in individual figure legends for each data set** |
|  |  | (*b*) Report category boundaries when continuous variables were categorized  **See results** |
|  |  | (*c*) If relevant, consider translating estimates of relative risk into absolute risk for a meaningful time period  **n/a** |
| Other analyses | 17 | Report other analyses done—eg analyses of subgroups and interactions, and sensitivity analyses  **Pages 17-19** |
| Discussion | | |
| Key results | 18 | Summarise key results with reference to study objectives  **Pages 20-25** |
| Limitations | 19 | Discuss limitations of the study, taking into account sources of potential bias or imprecision. Discuss both direction and magnitude of any potential bias  **Pages 24-25** |
| Interpretation | 20 | Give a cautious overall interpretation of results considering objectives, limitations, multiplicity of analyses, results from similar studies, and other relevant evidence  **Pages 24-25** |
| Generalisability | 21 | Discuss the generalisability (external validity) of the study results  **See abstract** |
| Other information | | |
| Funding | 22 | Give the source of funding and the role of the funders for the present study and, if applicable, for the original study on which the present article is based  **DAAD. This is an original study, the funders had no role in the study.** |

*Give information separately for exposed and unexposed groups.

**Note:** An Explanation and Elaboration article discusses each checklist item and gives methodological background and published examples of transparent reporting. The STROBE checklist is best used in conjunction with this article (freely available on the Web sites of PLoS Medicine at http://www.plosmedicine.org/, Annals of Internal Medicine at http://www.annals.org/, and Epidemiology at http://www.epidem.com/). Information on the STROBE Initiative is available at www.strobe-statement.org.
