# Supplementary figures and images for: Multivariable Regression Analysis in Schistosoma mansoni-Infected Individuals in the Sudan Reveals Unique Immunoepidemiological Profiles in Uninfected, egg+ and Non-egg+ Infected Individuals
Source: PLoS Negl Trop Dis. 2016 May 6;10(5):e0004629. doi: 10.1371/journal.pntd.0004629 (PMC4859533; doi:10.1371/journal.pntd.0004629)

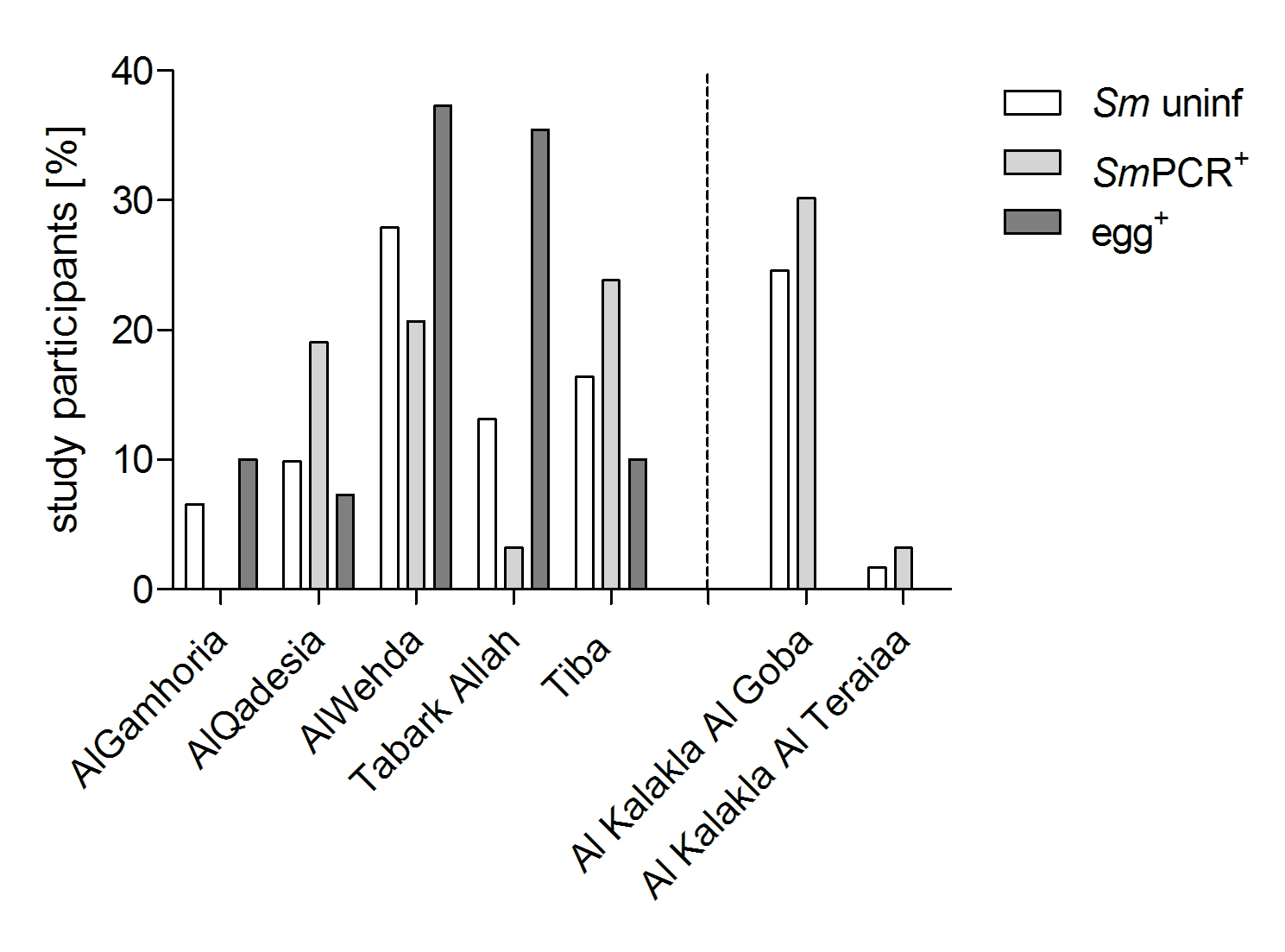

Supplement: S1 Fig — Study participants were grouped according to their villages which are located in the New Halfa Area (Kassala state) and along the White Nile in Khartoum state. (TIF) [file pntd.0004629.s002.tif]
