# Supplementary material for: Multivariable Regression Analysis in Schistosoma mansoni-Infected Individuals in the Sudan Reveals Unique Immunoepidemiological Profiles in Uninfected, egg+ and Non-egg+ Infected Individuals
Source: PLoS Negl Trop Dis. 2016 May 6;10(5):e0004629. doi: 10.1371/journal.pntd.0004629 (PMC4859533; doi:10.1371/journal.pntd.0004629)
Supplement: S1 Table — Number of participants within each group presenting further parasitic infections. N.B. Some participants were positive for more than one tested parasite. (DOCX) [file pntd.0004629.s003.docx]

**Supporting information**

**Supporting information Table 1**

|  | ***Sm* uninf (18/61)** | ***Sm*PCR^+^ (16/63)** | **egg^+^ (56/110)** |
| --- | --- | --- | --- |
| *Schistosoma haematobium* | 0 | 0 | 0 |
| Hymenolepis nana | 7 | 13 | 21 |
| Giardia lamblia | 10 | 14 | 26 |
| Entamoeba histolytica | 14 | 14 | 43 |
| Taenia saginata | 0 | 0 | 0 |
